# In Silico Methods for the Identification of Diagnostic and Favorable Prognostic Markers in Acute Myeloid Leukemia

**DOI:** 10.3390/ijms22179601

**Published:** 2021-09-05

**Authors:** Hande Yılmaz, Halil Ibrahim Toy, Stephan Marquardt, Gökhan Karakülah, Can Küçük, Panagiota I. Kontou, Stella Logotheti, Athanasia Pavlopoulou

**Affiliations:** 1 Izmir Biomedicine and Genome Center, Balcova, 35340 Izmir, Turkey; hande.yilmaz@med.uni-rostock.de (H.Y.); ibrahim.toy@msfr.ibg.edu.tr (H.I.T.); gokhan.karakulah@ibg.edu.tr (G.K.); can.kucuk@ibg.edu.tr (C.K.); 2 Izmir International Biomedicine and Genome Institute, Dokuz Eylül University, Balcova, 35340 Izmir, Turkey; 3 Institute of Experimental Gene Therapy and Cancer Research, Rostock University Medical Center, 18057 Rostock, Germany; stephan.marquardt@uni-rostock.de; 4 Department of Medical Biology, Faculty of Medicine, Dokuz Eylül University, Balcova, 35340 Izmir, Turkey; 5 Department of Computer Science and Biomedical Informatics, University of Thessaly, 35131 Lamia, Greece; pankontou@gmail.com

**Keywords:** acute myeloid leukemia, transcriptomics, clinical traits, bioinformatics, long-term survivors, minimal residual disease, diagnostics, prognostics

## Abstract

Acute myeloid leukemia (AML), the most common type of acute leukemia in adults, is mainly asymptomatic at early stages and progresses/recurs rapidly and frequently. These attributes necessitate the identification of biomarkers for timely diagnosis and accurate prognosis. In this study, differential gene expression analysis was performed on large-scale transcriptomics data of AML patients versus corresponding normal tissue. Weighted gene co-expression network analysis was conducted to construct networks of co-expressed genes, and detect gene modules. Finally, hub genes were identified from selected modules by applying network-based methods. This robust and integrative bioinformatics approach revealed a set of twenty-four genes, mainly related to cell cycle and immune response, the diagnostic significance of which was subsequently compared against two independent gene expression datasets. Furthermore, based on a recent notion suggesting that molecular characteristics of a few, unusual patients with exceptionally favorable survival can provide insights for improving the outcome of individuals with more typical disease trajectories, we defined groups of long-term survivors in AML patient cohorts and compared their transcriptomes versus the general population to infer favorable prognostic signatures. These findings could have potential applications in the clinical setting, in particular, in diagnosis and prognosis of AML.

## 1. Introduction

Acute myeloid leukemia (AML) is a complex and heterogeneous blood condition, and the most prevalent form of acute leukemia in adults, representing 32% of all adult leukemia cases [1]. The average age of AML diagnosis is 67 years [2]. AML is caused by the uncontrolled clonal proliferation of abnormal immature white blood cells or leukemic blasts. Since blasts accumulate in the bone marrow, peripheral blood, and other organs of the body as the disease progresses, the production of normal hematopoietic stem cells is prevented. If AML is not treated within one year of diagnosis, it progresses quickly, leading to excessive bleeding, life-threatening infections, infiltration of lymph nodes with leukemic cells, or metastases to distant organs [3,4,5].

In the standard practice, AML diagnosis and the decision on the suitable therapeutic strategy rely on the medical history and physical examination of each patient in combination with testing blood samples and bone marrow aspirates and biopsies. Laboratory tests for AML diagnosis include a series of procedures, such as a complete blood count and differential count, bone marrow biopsy and genetic testing, imaging or lumbar puncture, immunophenotyping, and cytogenetics [6]. AML patients are treated with intensive chemotherapy, targeted therapy, or bone marrow transplantation, which have overall improved survival outcomes, especially in younger patients [7]. However, despite the advances in the understanding of AML pathophysiology, mortality rates remain high. For example, in 2020, there were an estimated 60,530 new leukemia cases and 23,100 people died of this disease in the United States [8]. Long-term survival rates also appear to be low, and most older patients with AML die from disease relapse [9,10,11]. The poor outcomes reflect two major challenges in AML management. First, most cases arise without any detectable early symptoms and patients usually present with acute complications of bone marrow failure [12]. Second, complete hematologic remission (CR), which is a prerequisite for AML cure, cannot be readily achieved, since a significant percentage of AML patients eventually experience relapse after a seemingly successful treatment. This is because, although the therapeutic interventions can eliminate the majority of malignant cells, a few tumor cells inevitably evade therapy and remain persistently in the patient for years after the initial diagnosis [13]. This condition is termed minimal residual disease (MRD), and is a major cause of AML relapse [14]. These obstacles in AML treatment highlight the urgent need for identification of markers for early diagnosis, accurate prognosis, and estimation of CR.

The rapid progress in ‘-omics’ and high-throughput technologies has promoted the identification of novel genetic abnormalities and holds promise for accelerating the systematic characterization of AML cancer genomes. Gene expression profiling of AML patients has catalyzed the discovery of novel leukemia subgroups and of prognostic signatures [6]. The increasing availability of high-throughput data from blood and bone marrow of AML patients creates unprecedented opportunities for the comprehensive identification of diagnostic biomarkers of AML which could complement and update the existing ones [15,16,17]. Moreover, despite the poor outcomes of AML, there are a few fortunate cases that manage to survive over exceptionally extended periods of time, perhaps reflecting a remarkable resistance to MRD and disease progression. A novel rationale proposes that patients with exceptionally prolonged survival rates present distinct molecular profiles [18,19] that could be exploited to unveil favorable prognostic factors that differentiate them from patients with a more typical course of disease [20]. Understanding the biological determinants of survival in these extreme outliers may provide a route to improving responses in more typical patients, particularly if these studies identify new biomarkers to guide drug selection or novel pathways that are targetable. Seeking determinants of prolonged survival is particularly important in cancers with generally poor outcomes [20], such as AML. Taking these trends into account, in the present study, we applied integrative bioinformatics approaches to process, analyze, and interpret publicly available AML-relevant transcriptome data towards the identification of biomarkers for diagnosis and favorable prognosis of AML. Our diagnostic pipelines unveiled a signature of 24 differentially expressed genes, mainly involved in the cell cycle and immune response, that discriminate AML patients from normal controls and could be considered as candidate diagnostic biomarkers. It also enabled us to reveal a consensus group of genes that are consistently deregulated in survival outliers.

## 2. Results

**Figure 1:**
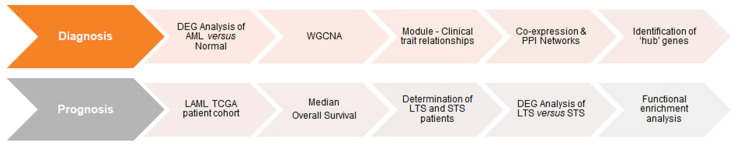
Flowchart diagram of the methodology employed in this study.

In this study, large-scale transcriptomic data were exploited towards the identification of genes that are differentially expressed between bone marrow AML samples and corresponding normal whole blood specimens. Given that genes with similar expression patterns (i.e., co-expressed genes) are usually implicated in similar biological processes, weighted gene co-expression network analysis (WGCNA) of the differentially expressed genes (DEGs) was performed [21], so as to identify modules (i.e., clusters of densely connected co-expressed genes) in AML with similar expression patterns. The genes contained in those modules were further correlated to AML clinical traits. Afterwards, different network-based methods were applied on the genes contained in these modules, so as to select the most biologically important genes/gene products. This analysis revealed twenty-four biologically important genes/gene products, which were further compared against two independent gene expression datasets. Furthermore, we set up a pipeline for the characterization of molecular signatures of AML long-term survivors (LTSs) that could enable prediction of MRD and survival probability. To this end, we estimated the median overall survival (OS) of the AML patients, and defined several groups of LTSs whose OS survival largely deviates from the median value. The transcriptomic profiles of the LTSs were compared to those of short-term survivors (STSs) to detect DEGs, and functional enrichment analysis was further performed to functionally interpret those genes. The overall procedure is briefly illustrated in Figure 1.

### 2.1. Identification of Diagnostic Markers in AML

#### 2.1.1. Comprehensive Characterization of Consensus AML Transcriptomes Compared to Normal Blood

**Figure 2:**
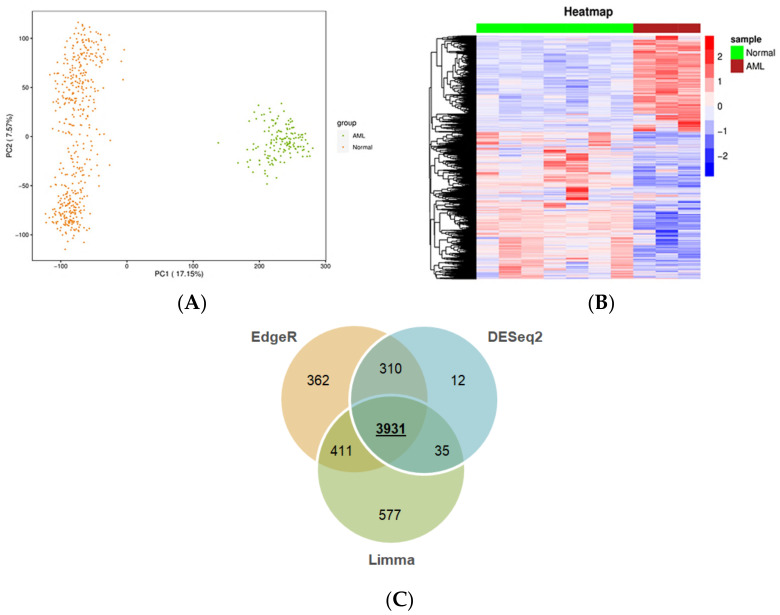
Genes differentially expressed between AML and normal samples. (**A**) Principal component analysis of TCGA and GTEx samples. Orange and green circles represent normal samples from GTEx and AML samples from TCGA, respectively. (**B**) Heatmap of the DEGs generated using DESeq2. Representative AML and normal samples are shown in maroon and green, respectively. Each row corresponds to a gene. The upregulated and the downregulated genes (based on Z-scoring) are shown in red and blue, respectively. (**C**) Venn diagram of the common and unique DEGs across edgeR, limma, and DESeq2.

First, we sought to identify the changes in transcriptomes of leukemic cells versus publicly available normal control samples, such as blood or bone marrow. It should be taken into account that unlike blood sampling, which is a fast and minimally invasive technique, bone marrow biopsy and aspiration are more distressing [6]. This issue poses limitations to obtaining a large biobank from this type of normal tissue, leading to inadequate numbers of relative high-throughput data in public platforms, such as GTEx and GEO. Nevertheless, encouraged by the adequate number (*n* = 456) of normal whole blood samples with available RNA-seq data in GTEx, we envisaged that comparing the transcriptome of malignant cells to that of normal blood holds a potential to reveal transcripts expressed in circulating leukemic cells. These could be considered as circulatory biochemical markers for blood-based diagnosis via real-time PCR, which is a routine and less painful clinical practice as opposed to other invasive diagnostic techniques. To this end, by using a uniform pipeline (described in the Materials and Methods), gene expression profiles from TCGA and GTEx, 151 AML and 456 normal blood samples, respectively, were compiled. The distribution of TCGA and GTEx samples according to PCA is shown in Figure 2A. As expected, AML and normal samples form discrete clusters, suggesting distinct transcriptomic profiles for the datasets from AML and normal whole blood samples. The statistically significant DEGs obtained by analyzing the RNA-seq data from TCGA-LAML and GTEx include 2056 upregulated, 2959 downregulated genes from edgeR (Figure S1); 1847 upregulated, 3108 downregulated genes from limma (Figure S1); and 1714 upregulated, 2575 downregulated genes from DESEq2 (Figure 2B). There are a total of 3931 common DEGs from edgeR, limma, and DESeq2 (Figure 2C), including 1503 upregulated and 2428 downregulated genes (Table S1). The protein-coding genes constitute 85% of those genes. A Venn diagram was generated using the function *VennDiagram* in the ‘VennDiagram’ package (v.1.6.20). The bar plot was created with the function *barplot* in the ‘graphics’ package (v.4.0.4).

#### 2.1.2. Weighted Gene Co-Expression Network Analysis of DEGs

##### Sample Clustering for Detecting Outliers

**Figure 3:**
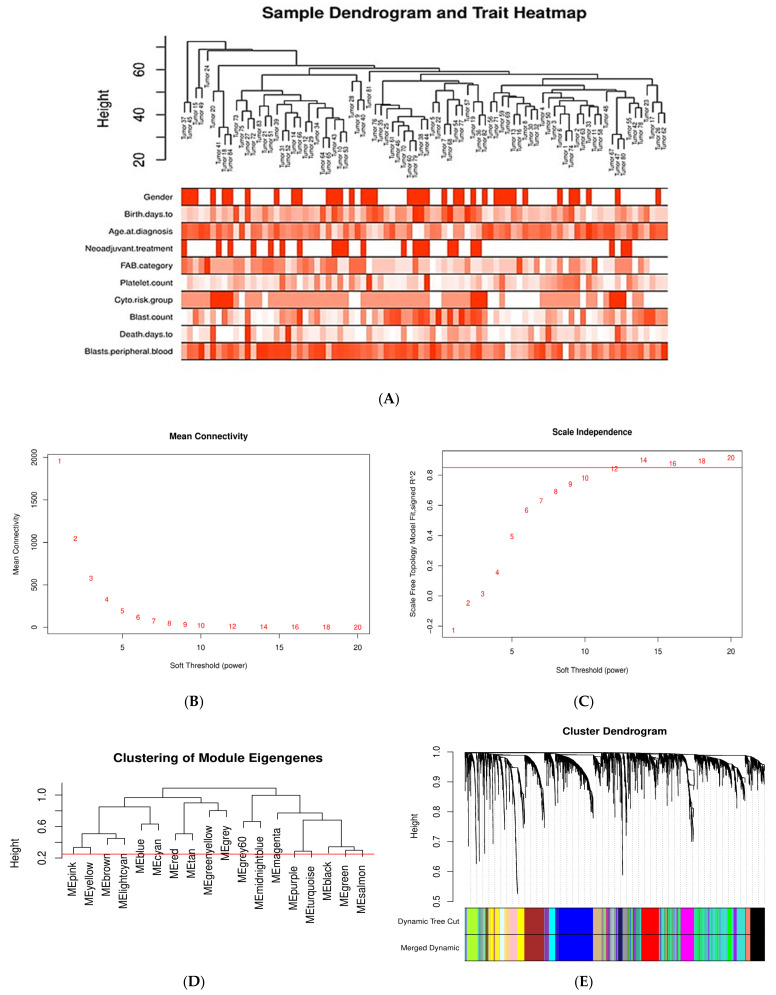

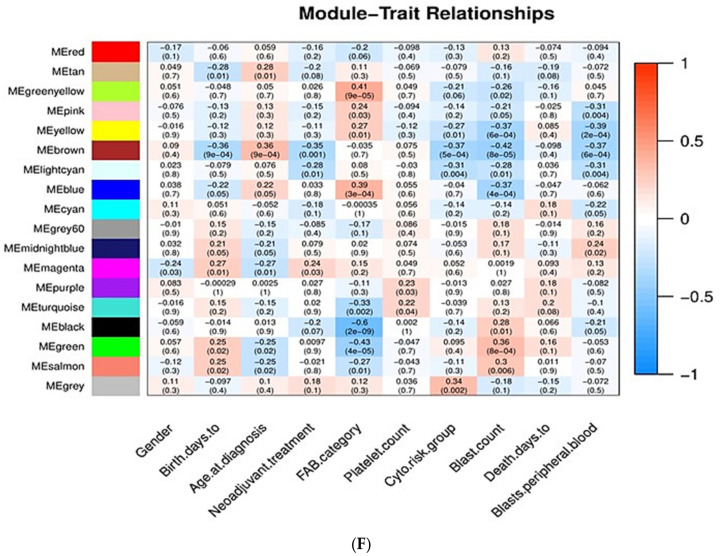
Sample clustering and module detection in WGCNA. (**A**) Sample dendrogram and trait heatmap for detecting outliers. The clustering was based on the expression data of the 3794 DEGs of the AML samples. All 84 samples are located within clusters. Darker shades of red indicate stronger positive correlation, whereas lighter shades of red denote weaker correlation. (**B**,**C**) Selection of soft-thresholding power (β) based on scale-free fit index and mean connectivity for various soft-thresholding powers. (**D**) Clustering of module eigengenes. The red line indicates cutoff height of 0.25. (**E**) Dendrogram of DEGs produced by hierarchical clustering based on dissTOM (1-TOM). Modules are represented by different colors. The two colored rows below the dendrogram represent the original and merged modules. (**F**) Module–trait correlation heatmap for AML clinical traits and gene module eigengenes. Each cell contains the Pearson correlation coefficient and *p* value. Blue indicates negative correlation, red denotes positive correlation.

The co-expression network of the DEGs in AML was constructed by WGCNA. First, outlier genes and samples were removed. In this way, a total of 3794 genes and 84 AML samples were used to construct the gene co-expression network. To determine whether all 84 AML samples were suitable for network analysis, the sample dendrogram and corresponding clinical traits were analyzed. All samples were included in the clusters and passed the cutoff thresholds (Figure 3A).

A soft-thresholding power (β) of 12 (scale free R^2^ = 0.8) was chosen by applying the approximate scale-free network topology criterion (Figure 3B,C). A dendrogram of DEGs was generated by average linkage hierarchical clustering using a TOM-based dissimilarity measure, by setting the cut height at 0.25 for module merging and the β value at 12 (Figure 3D,E). Gene modules are represented by different colors. The branches in the dendrogram represent modules, and each leaf, which is a short vertical line, corresponds to a gene. A total of 17 gene co-expression modules were determined, labeled as turquoise (735), blue (512), brown (308), yellow (277), green (258), red (238), black (223), pink (218), magenta (202), purple (186), greenyellow (139), tan (134), salmon (81), cyan (76), midnightblue (70), lightcyan (69), and grey60 (58); the numbers in the parentheses indicate the number of genes per module. The unclustered genes (10) were assigned to the gray module.

##### Associations of Gene Modules with Clinicopathological Traits of AML

The association between the 17 gene modules and ten important clinicopathological and molecular features of AML patients was investigated. The clinical significance of the selected traits is described below.

- Gender: Men are more prone to leukemia than women [22].
- Chronological age: Elderly people are more prone to AML than young people [23].
- Age at diagnosis: The incidence of AML is almost three times higher in adults aged 50–54 years and thirteen times higher in those aged 70–74 years as compared to adults aged 20–24 years [24,25].
- Neoadjuvant treatment: Whether patients have previously received chemotherapy or not; patients who have previously received chemotherapy are more prone to AML [26].
- FAB category: Based on the type of cell from which leukemia developed and the degree of maturity of these cells. FAB category corresponds to the French–American–British classification scheme which is useful for classifying AML into subtypes from M0 (undifferentiated AML or AML with minimal differentiation) to M7 (acute megakaryoblastic leukemia, AMegL) [27].
- Platelet count: AML is characterized by low platelet count. Normal human blood includes 150,000–450,000 platelets per microliter. Platelet count decreases in AML [28,29].
- Cytogenetic risk group: Cytogenetic analysis is recognized as being the most important prognostic indicator in acute myeloid leukemia. According to the cytogenetic risk group, AML is divided into three groups: favorable risk, intermediate risk, and unfavorable risk. Cytogenetic tests help predict the response of cancer to treatment and allow physicians to design a more effective therapy [30,31].
- Blast count and peripheral blood blast: For the diagnosis and classification of AML, the percentage of peripheral blood (PB) and bone marrow (BM) blasts is especially important. BM blasts normally represent 1% to 5% of marrow cells. Generally, a percentage of 20% blasts is required for AML diagnosis. Most patients with AML have a higher percentage of BM blasts compared to PB blasts [32,33,34].
- Days to death: Related to overall survival, which is the survival time after initial diagnosis. Measuring the overall survival is required to assess how well a new treatment works in a clinical trial. In AML, the 5-year overall survival is less than 50%; regarding elderly patients, only 20% survive 2 years after diagnosis [35,36].

Pearson correlation coefficients (*r*) between modules’ eigengenes and the clinical traits were calculated to detect important module–trait relationships. In our study, those modules with |*r*| > 0.25 and *p* value < 0.05 were considered to be statistically significant. As shown in Figure 3F, twelve modules (tan, greenyellow, pink, yellow, brown, lightcyan, blue, magenta, turquoise, black, green, and salmon) are significantly associated with clinical traits. The genes from each of those modules were selected for further analysis.

#### 2.1.3. Reconstruction of AML Molecular Networks

**Figure 4:**
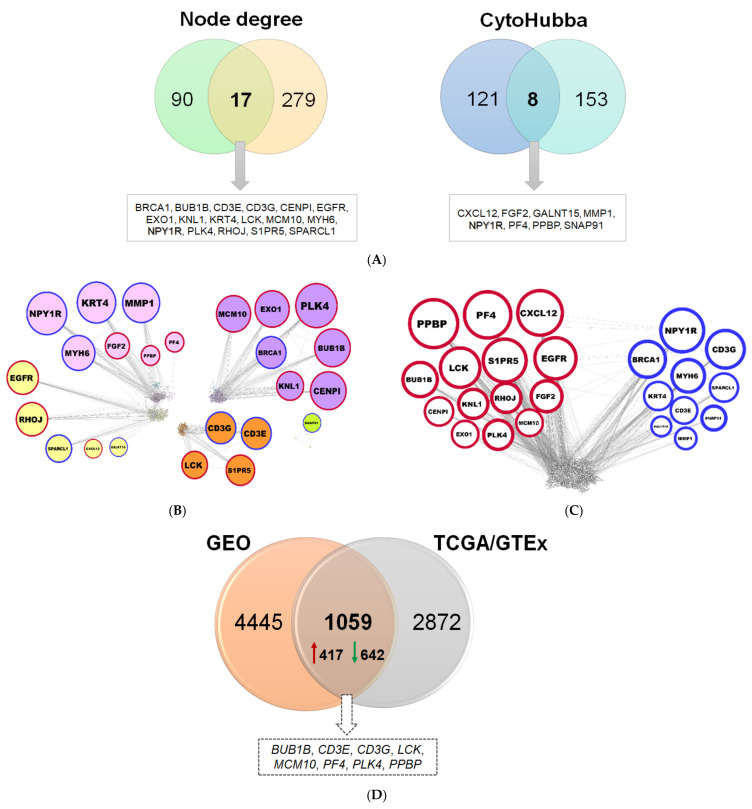
Important nodes identified through gene co-expression and PPI network. (**A**) Venn diagrams depicting the common nodes between the co-expression and PPI networks according to node degree and cytoHubba, respectively. (**B**) Co-expression network showing the 24 common genes, as distributed in pink, magenta, yellow, brown, and greenyellow modules. The nodes correspond to genes and the edges (connecting lines) represent the associations among genes. The size of the nodes is proportional to the number of their immediate connections (degree of connectivity). Red and blue border lines indicate up- and downregulation, respectively. (**C**) PPI network with the 24 common proteins. (**D**) Venn diagram for validation of diagnostic signatures, showing the common DEGs between the TCGA/GTEx and the two merged GEO datasets.

The 3154 genes belonging to the twelve modules (Figure 3E) were used to construct co-expression and PPI networks. In the co-expression network, 90 genes with ≥17 connections were identified, and the top 121 genes were selected through cytoHubba by combining the output of eleven different algorithms (Figure 4A). Likewise, in the PPI network, 279 hubs and 153 important protein nodes were identified (Figure 4A). The nodes that correspond to genes or gene products, found to be common between co-expression and PPI networks, based on degree of connectivity and cytoHubba, are shown in Figure 4A. NPY1R was detected by using both approaches (Figure 4A). In this way, a total of 24 genes were found, of which *PPBP*, *PF4*, *CXCL12*, *BUB1B*, *LCK*, *S1PR5*, *EGFR*, *CENPI*, *KNL1*, *RHOJ*, *FGF2*, *EXO1*, *PLK4*, and *MCM10* genes are upregulated, and *NPY1R*, *BRCA1*, *CD3G*, *MYH6*, *KRT4*, *SPARCL1*, *CD3E*, *GALNT15*, *MMP1*, and *SNAP91* genes are downregulated (Figure 4B,C).

#### 2.1.4. Comparison of Diagnostic Signatures with Independent AML Datasets

Those genes found to be differentially expressed between the TCGA AML and corresponding normal GTEx samples were further compared against two independent datasets obtained from NCBI GEO. The number of DEGs detected between AML and control (i.e., from matched healthy donors) samples for each NCBI GEO dataset is 793 (GSE138702) and 4780 (GSE9476) (Table S1), by applying stringent criteria. The DEGs found in each of the two transcriptome datasets were merged and the duplicates were removed; collectively, 5504 genes were compiled (Table S1). A rather significant overlap was found between the DEGs from TCGA/GTEx and those from the two GEO datasets; in particular, 1059 genes were found in common (representing 27%), eight of which constitute hubs (33%) (Figure 4D).

### 2.2. Inferring Favorable Prognostic Markers from the Transcriptomic Profiles of Long-Term Survivors of AML

Even after a seemingly successful treatment, a significant percentage of AML patients is at risk of disease recurrence [14], as a consequence of minimal residual disease. It has been estimated that of those patients who will achieve initial remission, approximately 50–70% of them will relapse within three years. Nevertheless, a lower percentage of adult AML patients survive to five years [37] while some infrequent cases with survival over ten years have also been reported [38]. In general, across several cancer types, there are rare cases of patients who manage to remain progression-free for atypically long durations of time, while in some rare cases patients never relapse [20,39]. Intriguingly, tumors of these unusual patients present distinct genetic and gene expression profiles as compared to the patients with a more typical course of disease [18,19]. Patients with exceptionally prolonged survival rates comprise a rare, but distinct, clinical subpopulation which holds the potential to reveal signatures of favorable prognostic factors that could be informative for the effective management of the general population of cancer patients [20]. According to these notions, the patients with unusually prolonged survival (hereafter termed long-term survivors, LTSs) in our study cohort might plausibly better cope with MRD and relapse and, therefore, their distinct transcriptomes, as compared to the general population, can be used to provide prognostic signatures associated with resistance to MRD.

**Figure 5:**
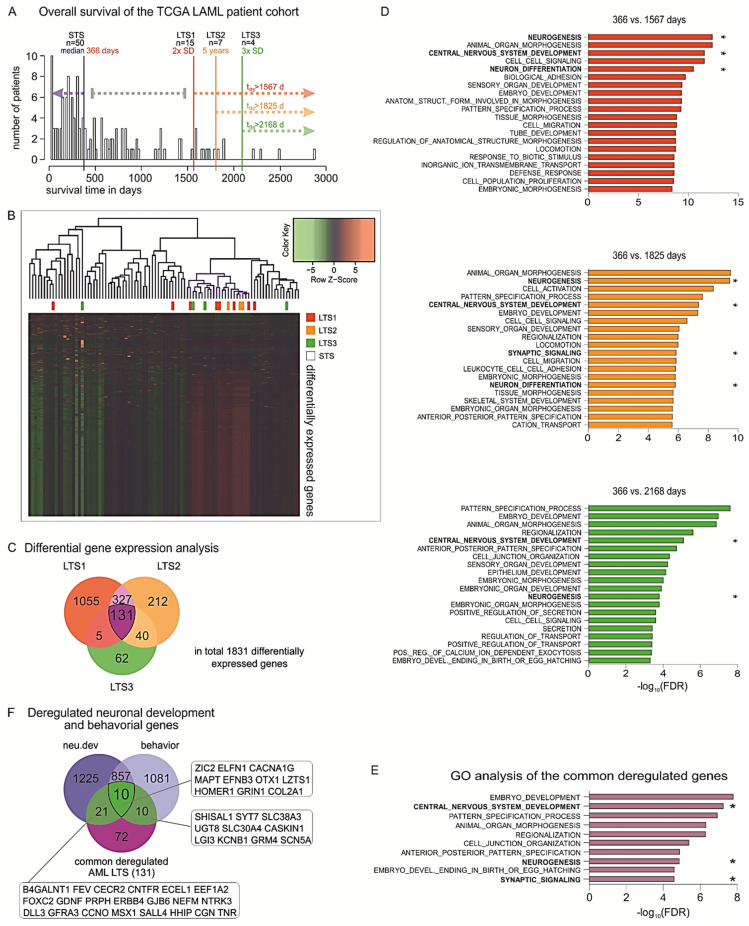
Identification of favorable prognostic factors based on transcriptomic profiles of LTSs. (**A**) Histogram of the overall survival (OS) of the TCGA LAML cohort with median (366 days, *n* = 50) and cutoffs for the three long-term survivor (LTS) groups: 2 × SD = 1567 days (*n* = 15), 5-year survival = 1825 days (*n* = 7), and 3 × SD = 2168 days (*n* = 4). (**B**) Heatmaps of the DEGs in the three LTS subpopulations versus the STSs. (**C**) Venn diagram of the deregulated genes of all three analyses showing large overlaps between analyses and 131 genes in common. (**D**) GSEA of GO biological functions associated with the DEGs in each LTS subgroup versus the patients with OS < 366 days. (**E**) Top 10 enriched GO biological functions for the 131 genes that are commonly deregulated across all LTS subgroups, ranked by order of significance (−log10 of FDR value). (**F**) Overlapping of the 131 genes with previously identified nervous system-related genes showed that 31.3% of these genes are indispensable for neuronal development, neurological function, or both (gene abbreviations are indicated in the respective boxes). * Nervous system-related pathways.

To this end, in the cohort of AML patients of TCGA, we distributed the patients according to their OS and estimated the median OS which would reflect the typical course for the disease for the specific cohort. The median OS for the whole AML population was 366 days (*n* = 50 patients), while the standard deviation value was estimated as 600.5 days. Then, we defined populations of patients with exceptionally favorable prognosis within the study cohort in a time-dependent manner as the patients who survive i) longer than the median OS + 2 SD (>1567 days, *n* = 15 patients), or ii) longer than the median OS + 3 SD after initial diagnosis (2168 days, *n* = 4 patients). Given that in clinical practice the patients who are alive 5+ years past diagnosis are considered LTSs [40], we additionally included the patients who survive past this clinical milestone (1825 days, *n* = 7 patients) in our analysis (Figure 5A). We sought to identify the distinct tumor gene expression profiles across these LTSs versus those of the patients who are deceased by 366 days (Figure 5B). Our analysis revealed common deregulated genes between all three comparisons (Figure 5C). Subsequent gene set enrichment analysis (GSEA) showed that biological functions such as locomotion, cell migration, and cell junctions are enriched in the DEGs in each LTS subgroup. This is expected, since these traits are linked with a propensity of cancer cells to metastasize. Surprisingly, among the top significantly enriched processes, we also observed deregulations of genes that are involved in developmental processes, in particular neurogenesis, central nervous system development, neuron differentiation, and pattern specification (Figure 5D). We further identified a group of 131 DEGs that remain consistently deregulated in all three groups and, hence, could be important for maintaining survival over extended periods of time (Table S2).

Strikingly, GSEA showed that the commonly deregulated genes are strongly involved in early and late stages of embryonic development, and particularly in neuronal development and neurological function. In detail, the top 10 most significantly enriched biological processes were embryo development, central nervous system (CNS) development, pattern specification process, animal organ morphogenesis, regionalization, cell junction organization, neurogenesis, anterior–posterior pattern specification, synaptic signaling, and embryo development ending in birth or egg hatching (Figure 5E). Among the transcripts found upregulated in LTSs and involved in many of these processes were glial cell-derived neurotrophic factor (GDNF), a neurotrophic molecule that promotes neuronal differentiation and dopaminergic development [41], as well as several products of homeobox genes that are master regulators of development, morphogenesis, and cell differentiation, for example, distal-less homeobox 1 (DLX1), msh homeobox 1 (MSX1), and orthodenticle homeobox 1 (OTX1) [42]. We further considered the cytogenetic background of the patients of the LTS and STS subgroups in regard to the reciprocal translocation t(15;17), which is associated with decreased incidence of relapse and improved survival upon all-trans retinoic acid (ATRA)-containing treatments. In particular, six patients across the LTS subgroups versus six patients in the STS subgroup bear a t(15;17) translocation, leading to fusion of the promyelocytic leukemia gene (PML) to retinoic acid receptor alfa (RARA), which is specifically sensitive to ATRA and arsenical derivatives [43]. To eliminate survival bias in the LTSs caused by this cytogenetic feature, we excluded these patients and performed the comparisons between the transcriptomes of the STSs and LTSs. Strikingly, we found that the DEGs in LTSs remained significantly associated with CNS development, neuronal development, neuronal differentiation, and neurogenesis.

In view of these findings, we sought to further explore the potential link between long-term survival and the nervous system. We have recently performed a comprehensive, phenotype-driven identification of nervous system-related genes. These include all protein-coding genes that are essential for normal nervous system development (neu.dev) and neurological function (behavior), whereby their knockout leads to malformations of the neural system components or behavioral disorders, respectively. Using system-based approaches, we had found 1246 genes that are specific for neuronal development (neu.dev) and 1086 for neurological function (behavior), while 863 genes are involved in both processes [44]. Overlapping of the ‘neu.dev’ and ‘behavior’ lists of genes with the 131 commonly deregulated genes in LTSs showed that, overall, 41 (31.3%) of them are nervous system-related genes, whereby 21 are indispensable for neuronal development, 10 for neurological function, and 10 for both processes (Figure 5F). Overall, our analyses indicate a strong association of the nervous system with the exceptionally prolonged survival and patient outcomes in AML.

## 3. Discussion

The incidence of AML increases with age, and the survival rate is exceptionally poor when diagnosed after age 65. In the present study, we employed integrative bioinformatics approaches on AML-relevant transcriptomic data to identify biomarkers of diagnosis and favorable prognosis.

We identified a set of 24 hub genes which discriminate AML cells from normal controls and could be valuable as a diagnostic signature. As expected, several of these genes (*BRCA1*, *BUB1B*, *CENPI*, *EXO1*, *KNL1*, *MCM10*, and *PLK4*) regulate genomic stability and cell proliferation. DNA damage repair-associated *BRCA1* is a known tumor suppressor gene, essential for DNA synthesis and cellular replication [45]. Mitotic checkpoint serine/threonine kinase B (BUB1B) is a vital component of the mitotic checkpoint and it is necessary for normal mitosis progression [46,47]. Centromere protein I (CENPI) is a structural component of the kinetochore, required for timely progression through G2 phase, mitosis, and chromosome stability [48,49]. Exonuclease I (EXO1) contributes to the regulation of the cell cycle checkpoints, the maintenance of the replication fork, DNA repair, and genomic instability [50,51]. Kinetochore scaffold 1 (KNL1) is essential in spindle assembly checkpoints, chromosome segregation, and kinetochore–microtubule attachments [52,53]. Minichromosome maintenance 10 replication initiation factor (MCM10) is required for DNA synthesis and entry into or ending of S-phase [54,55]. Polo-like kinase 4 (PLK4) plays a crucial role in controlling centriole duplication, and its deregulation causes centrosome number abnormalities, mitotic defects, chromosomal instability, and, consequently, tumorigenesis [56]. Genomic instability and cell cycle deregulation favor the rapid proliferation and clonal expansion of cells harboring oncogenic DNA alterations in AML [57,58,59]. The upregulation of hub genes controlling these processes reflects the tendency of hematopoietic precursor cells in bone marrow to accumulate DNA lesions and increase their proliferation rate at the expense of normal hemopoiesis, resulting in bone marrow failure.

Notably, several genes of the diagnostic signature are involved in immune and inflammatory responses. For example, the CD3e molecule (CD3E) and CD3g molecule (CD3G) form a complex with TCR, a T cell receptor that plays an important role in T cell antigen recognition and signal transduction. Decreased expression of *CD3E* and *CD3G*, as in our study, leads to deregulation of T cell function [60]. Moreover, C-X-C motif chemokine ligand 12 (CXCL12) and its receptor CXCR4 are key elements for the adaptive and innate immune response and also for bone marrow organization and maintenance [61]. They are largely responsible for hematopoietic stem cell migration, homing, and survival in bone marrow [61]. CXCL12, found to be upregulated in our study, facilitates trafficking and homing of leukemic cells to the bone marrow microenvironment [62]. Furthermore, we identified the platelet-related genes *platelet factor 4* (*PF4*) and *pro-platelet basic protein* (*PPBP*) as components of the AML diagnostic signature. In general, platelets are anucleate cell fragments known for their central role in coagulation and vascular integrity and their deregulation in AML patients. Beyond their typical roles as fragmentary mediators of hemostasis and thrombosis, platelets orchestrate diverse immunological processes, including fighting against microbial threats, recruitment and promotion of innate effector cell functions, modulating antigen presentation, and enhancement of adaptive immune responses [63,64]. They usually interact with blood cells, as well as circulating tumor cells, via adhering or releasing a variety of soluble mediators, which can modulate the activity of hematopoietic stem and progenitor cells. Such platelet-coated tumor cells not only have an enhanced ability to attach to endothelial cells and endothelial extracellular matrix and thus promote tumor invasion and metastasis, they are also protected from the clearance of immune surveillance [65]. Overall, these findings underscore an implication of the immune system in AML pathophysiology which could be particularly relevant for the prediction of AML patients with favorable responses to immunotherapy. In particular, immunotherapy has catalyzed a paradigm shift in cancer treatment and has become the standard of care for several solid tumors. As our understanding of the genomic landscape of AML moves forward and the arsenal of immunotherapies is being enriched, their extension to AML represents an attractive clinical option, but this endeavor requires the establishment of guidelines for the prediction of groups of patients who would most benefit from immune targeting. A recent study on primary bone marrow samples from independent cohorts of AML patients defined immune-infiltrated and immune-depleted disease classes, revealed critical differences in immune gene expression across age groups and molecular disease subtypes, and provided compelling evidence that immune landscapes predict chemotherapy resistance and immunotherapy response [66]. The immune contexture is an emerging parameter of AML pathophysiology, and further investigation of the tumor microenvironment (TME) of the bone marrow, in combination with identification of immunomodulatory biomarkers in the blood, may allow molecular predictions of immunotherapeutic benefit and guide personalized treatment decisions. Further studies can uncover the molecular mechanisms underlying AML immunobiology and their translation to therapeutic solutions.

The asymptomatic nature of AML in combination with MRD and frequent disease relapse hamper AML prognostics, but a few patients manage to remain progression-free for exceptional durations of time. Despite their sparsity, unusual patients can inform cancer biology [20]. For example, families with rare and high penetrant germline mutations (Li–Fraumeni syndrome) have catalyzed understanding of the function of tumor suppressor genes [67], while populations with diminished cancer incidence (subjects with Laron syndrome in Ecuador) provided insights on cancer metabolism and the insulin-like growth factor (IGF) pathway in tumor progression [68]. In an analogous manner, cases with exceptionally prolonged survival may bear distinct molecular characteristics, the elucidation of which could ultimately improve the outcome of individuals with more typical disease trajectories [20]. Long-term survivorship has, at least in part, a genetic and immunological basis [18,19,69]. Molecular factors contributing to favorable survival in a patient, who may otherwise have had a more typical course of disease, may include germline genetics, endogenous antitumor immune responses, tumor mutational load, and characteristics of the microenvironment [20]. Putting this novel rationale under testing, we defined subgroups of LTSs in our study population and investigated their distinct transcriptomic profiles as compared to patients below the median OS in order to infer favorable prognostic signatures which could be associated with resistance to disease progression. Surprisingly, this approach revealed that malignant cells of LTS AML patients exhibit prominent deregulations of genes involved in neuronal processes, thereby providing hints for associations between exceptionally prolonged survival and neurogenic cell traits.

The effect of the nervous system on tumor progression has recently come into the spotlight as an enigmatic phenomenon with a paramount effect in disease outcome. We recently demonstrated that all cancer types can activate neuronal networks ‘off-context’ and this ability largely influences the acquisition of aggressive characteristics. This trend seems to have a universal character across several cancers, since nervous system-related gene transcripts are upregulated in invasive stages and predict clinical outcomes in all cancer types, including AML. The prognostic potential of each nervous system-related gene depends on the cancer type [44]. Depending on the nervous system-related genes that are ectopically activated in a cancer cell context, patient survival can be affected either positively or negatively. Furthermore, different cancer types co-opt genes essential for the development of distinct anatomical parts of the nervous system. In the case of AML, genes indispensable for the somatic sensory system, the cerebellum fissum, the sympathetic nervous system, and the dentate gyrus granule cell layer are associated with AML patient outcomes [44]. Herein, we show that deregulations of nervous system-related genes are strongly associated with exceptionally long survival periods in AML. The relevant underlying mechanism awaits to be unveiled. On the one hand, it has been recently described that leukemia stem cells can evade the arachnoid space, migrate, proliferate in the cerebrospinal fluid (CSF), and consequently infiltrate perivascular spaces and brain parenchyma. The CNS is an immune-privileged site that can protect leukemic cells from chemotherapy. Overexpression of the neural cell adhesion molecule NCAM/CD56 in the surface of leukemic stem cells increases their ability to infiltrate the CNS [70]. In this regard, deterministic deregulation of selected neuronal signaling pathways may hypothetically enable leukemic cells to ‘mingle’ within the neuronal tissue microenvironment and rewire with the electrically active neurons [70]. On the other hand, Hanoun et al. (2014) demonstrated that adrenergic signals regulate AML and that when leukemic cell injections in mice are followed by sympathetic denervation, the course of disease is accelerated. They further provided compelling evidence that sympathetic neuropathy represents a mechanism by which AML co-opts the microenvironment to its own advantage in order to deplete niche cells that maintain healthy hematopoietic stem cells and expand leukemia-supportive mesenchymal progenitors [71]. The interactions between leukemic and neuronal cells in the context of AML recurrence, MDR, and/or metastasis remain a *terra incognita*. Future research can unveil how ‘hijacking’ of networks of the nervous system might offer several selective advantages in leukemic cells, especially those that persist in the circulation after initial AML management, and represent the ‘seeds’ of MRD. Conversely, it would be interesting to investigate whether activation of other neuronal signaling pathways can be protective against MRD in AML patients. Deciphering the neuronal signaling pathways that are beneficial for leukemic cell survival over those with a detrimental effect is anticipated to shed more light on AML pathophysiology and might have important translational implications in disease prevention and management. Such studies could open up novel avenues for therapeutic interventions, for example, by repurposing already approved neurological drugs to target the cancer–neuronal crosstalk and prevent progression to aggressive stages [72].

Last, but not least, the profiles of the rare LTS patients who either never regress or manage to remain relapse-free for extended periods of time might be particularly meaningful in the context of AML prognosis. Their alleged resistance to disease progression or relapse could be an indicator of better abilities to cope both with MRD, which may be associated not only with epidemiological, environmental, or lifestyle factors, but also with their inherent molecular traits [20]. Hints supporting the influence of the hereditable genetic characteristics on the resistance to recurrence arise from studies demonstrating that ethnicities with inherently different genetic backgrounds, such as Caucasians and African Americans, exhibit differences in the AML prognosis, whereby Caucasian males have better CR and overall survival than African American males [73]. It would be interesting to investigate whether patients with unusually long survival periods share favorable intrinsic characteristics that overall predispose them to better detect and self-eliminate residual cancer cells during or after treatment. Checking for additional associations between the inherent characteristics of the LTSs and several clinical traits that are important for treatment personalization, such as cytogenetic characteristics and therapy regimens, could also be highly informative for the management of AML. This endeavor would require appropriate study designs that are specifically focused on LTS patient subgroups, where patients surviving over 5, 10, or more years will be oversampled and compared to those with more conventional disease trajectories [20]. Unveiling the cancer genomes and intrinsic genetic traits of this novel clinical population as opposed to relapsing cancer patients or even the general non-cancer population could reveal predisposing factors for resistance to recurrence. Future clinical trials could aim at the characterization of the molecular factors that are associated with LTSs. Such clinical study endpoints would be invaluable for the prognosis and management of AML and could enable not only the identification of patients with unfavorable prognosis who will need special treatments, but also the design of appropriate targeted strategies that prolong survival.

## 4. Materials and Methods

All analyses were performed in the R statistical computing environment v.4.0.3 (https://www.r-project.org, accessed on 1 September 2020) [74], unless otherwise stated.

### 4.1. Data Retrieval, Processing, and Analysis

**Figure 6:**
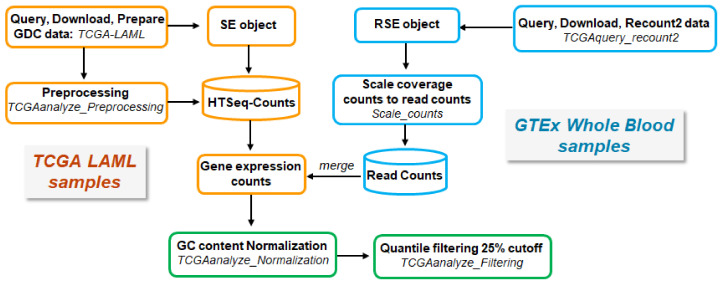
Schematic workflow of retrieving, processing, and merging TCGA and GTEx data.

Non-normalized, raw RNA-seq read count data of AML tissue samples were downloaded from The Cancer Genome Atlas (TCGA) using the ‘TCGAbiolinks’ R/Bioconductor package [75]. Since there are no available matched normal tissue samples for AML in TCGA, raw RNA-seq read counts per gene for whole blood normal tissue samples were downloaded from the Genotype-Tissue Expression (GTEx) Portal (https://gtexportal.org/home/, accessed on 14 September 2020) using the ‘TCGAbiolinks’ package. The RNA-seq data were generated from two different technological platforms (TCGA and GTEx), and due to their inherent variability, they had to be processed in a uniform way in order to be comparable. To ensure comparability across TCGA-derived AML and GTEx-derived whole blood normal tissue samples, all raw RNA-seq read counts were normalized and filtered, as suggested by the original TCGAbiolinks workflow [76,77] The overall procedure is illustrated in (Figure 6).

#### 4.1.1. RNA-seq Data Acquisition and Processing

##### AML Samples from TCGA

Acute myeloid leukemia (LAML) harmonized data (hg38) (Illumina HiSeq platform) were obtained from the Genomic Data Common (GDC) Portal (https://portal.gdc.cancer.gov/, accessed on 14 September 2020) of the TCGA database. GDC data were queried, downloaded, and prepared using the functions *GDCquery, GDCdownload,* and *GDCprepare,* respectively. A total of 151 samples including expression data of primary blood-derived cancer—peripheral blood were analyzed. Samples lacking clinical information were excluded from our study, and therefore 84 tumor samples were included. Pre-processing steps were applied according to the TCGA’s workflow as recommended in the ‘TCGAbiolinks’ package in order to find possible outliers in transcriptome data using the *TCGAanalyze_Preprocessing* function. This function generates a count matrix ready to be provided as input in the downstream analysis pipeline.

##### Normal Tissue Samples from GTEx

Collectively, 456 of normal whole blood GTEx samples were downloaded through the Recount2 project using the function *TCGAquery_recount2* in the ‘TCGAbiolinks’ package, as ranged summarized experiment (RSE) objects. The raw counts were scaled with the *‘scale_counts’* function in the ‘Recount’ package (v.1.16.1) [78,79].

##### Processing and Merging of TCGA- and GTEx-Derived Data

HTSeq counts from TCGA and read counts from GTEx were merged and gene expression counts were acquired, which were normalized for ‘GC content’ using the function *TCGAanalyze_Normalization* of the EDASeq protocol for exploratory data analysis (EDA) [80] in the ‘TCGAbiolinks’ package. Quantile filtering was applied with a cutoff of 25%.

##### Principal Component Analysis

Principal component analysis (PCA) of the TCGA and GTEx datasets was employed to detect batch effects. Raw RNA-seq read counts were converted to counts per million reads mapped (CPM) values with the function *cpm* in the ‘edgeR’ package (v.3.32.1) [81]. The functions *prcomp* and *ggplot* in the ‘ggplot2′ package (v.3.3.3) [82] were used to create a PCA plot.

### 4.2. Identification of Differentially Expressed Genes

To identify those genes that are differentially expressed between AML and normal samples, with the same direction (i.e., up- or downregulated), three different methods implemented in the R packages edgeR (v 3.32.1) [81], limma (v 3.44.3) [83], and DESeq2 (v.1.28.1) [84] were used.

Normalization by trimmed mean of M values (TMM) was performed with the *calcNormFactors* function in the ‘edgeR’ package (v.3.32.1). The negative binomial (NB) distribution was used to model the RNA-seq read counts per gene per sample in edgeR. Then, estimating dispersion was calculated with the *estimateDisp* function. DEG analysis between the two RNA-seq groups (i.e., AML versus normal) was performed using likelihood ratio tests (LRTs) including the *glmFit* and *glmLRT* functions in edgeR.

The *Voom* function in the ‘limma’ package was utilized to transform RNA-seq count data to log2 counts per million (logCPM). The *lmFit* and *contrasts.fit* functions in the ‘limma’ package were utilized for linear modeling. Next, the *eBayes* function in the ‘limma’ package was used to carry out empirical Bayes moderation by borrowing information across all genes, so as to obtain more accurate estimates of variability across genes. The DEGs were extracted by using the *decideTests* function.

The package ‘DESeq2′ uses NB generalized linear models including the estimates of dispersion from the edgeR method. The standard differential expression analysis was performed using the function *DESeq.* Result tables were generated with the function *results*.

For detecting statistically significant differentially expressed genes, the threshold for the absolute log_2_ fold change (FC) was set at two (|log_2_FC ≥ 2|), and the adjusted *p* value by the Benjamini and Hochberg false discovery rate (FDR) method [85] less than 0.05.

To visualize DEGs, heatmap, MA and volcano plots were created. In particular, the *EnhancedVolcano* function in the R package ‘EnhancedVolcano’ (v.1.8.0) was utilized to generate volcano plots of the DEGs. Heatmap plots of logCPM values were created with the *pheatmap* function in the ‘pheatmap’ package in R (v.1.0.12). The *plotMD* function in the R package ‘limma’ was used to generate MA plots of the DEGs.

### 4.3. Construction of Weighted Gene Co-Expression Network

The common DEGs from the 84 AML samples were used to construct a gene co-expression network with the package WGCNA [21] (v.1.69) implemented in R. To this end, the FPKM values of the common genes were downloaded from the GDC Portal (https://portal.gdc.cancer.gov/; accessed on 10 December 2020) of the TCGA database and processed as described earlier (Section 4.1.1); analysis was conducted using the log2 (fpkm + 1) value. The *goodSamplesGenes* function was used to iteratively filter genes and samples.

The soft-threshold power was applied to construct pairwise Pearson correlation matrices so as to measure gene co-expression similarities. The function *pickSoftThreshold* was used to analyze network topology and select a suitable soft-thresholding power. The adjacency matrices were calculated with the function *adjacency*, which converts the Pearson correlation coefficients into gene connection strengths. The adjacency matrices were transformed into a topological overlap matrix (TOM) with the function *TOMsimilarity* in order to minimize the effects of noise. To group genes into modules, the average linkage hierarchical clustering method was applied by using the function *hclust* to cluster module eigengenes (i.e., first principal component) with a TOM-based dissimilarity measure; a relatively high minimum module size of thirty (minModuleSize = 30) was selected. The *dynamicTreeCut* algorithm was applied to detect modules with an eigengene correlation coefficient above 0.75, by setting the cut height threshold (MEDissThress) at 0.25.

### 4.4. Identification of Clinically Important Modules

Significant clinical data including gender, days to birth, age at diagnosis, history neoadjuvant treatment, French–American–British (FAB) category, platelet count preresection, cytogenetic risk group, blast count, days to death, and percentage of blasts in peripheral blood were acquired from the GDC Portal (https://portal.gdc.cancer.gov/, accessed on 14 September 2020). GDC data were processed as described earlier (Section 4.1.1).

To determine whether all AML samples were suitable for network analysis or to detect any outliers in these samples, the clustering dendrogram of the samples and the corresponding clinical traits was generated using the *hclust* function in the ‘WGCNA’ package based on their Euclidean distance.

### 4.5. Co-Expression and Protein–Protein Interaction (PPI) Network Analysis

Given that the modules detected by WGCNA are usually large, consisting of many genes, it is important to determine which genes are more representative of each module, as well as of the entire network. In the biological networks, the most highly connected genes, ‘hub’ genes, are generally considered to be of higher biological significance and more relevant to the overall function of the network [86,87]. The intra-modular hubs are central to a given module, whereas inter-modular hubs are intermediate between two or more modules.

To this end, the genes contained in the modules associated with AML clinical traits were used to construct a gene co-expression network; genes with a Pearson correlation coefficient more than 0.3 were selected. Furthermore, those genes were provided as input to the Search Tool for the Retrieval of Interacting Genes (STRING) (v.11.0) (https://string-db.org/, accessed on 1 February 2021) database [88] in order to investigate the functional and/or physical associations among their corresponding gene products. A highest confidence score of ≥0.9 was set as a cutoff.

The topological properties of the gene co-expression and protein–protein interaction (PPI) networks were further investigated through Cytoscape (v.3.8.2) (https://cytoscape.org/, accessed on 12 February 2021) [89], an open-source platform for network visualization and manipulation. In both networks, those nodes with high connectivity [90,91], that is, connected to more than 17 immediate neighboring nodes, were selected. Moreover, the Cytoscape plugin cytoHubba [92], which provides eleven local and global methods for network topological analysis, was utilized to select the top thirty nodes (for each of the 11 methods).

### 4.6. NCBI GEO Gene Expression Datasets

The publicly accessible repository NCBI GEO DataSets (https://www.ncbi.nlm.nih.gov/gds/, accessed on 20 February 2021) [93,94] was searched thoroughly for transcriptomic datasets using the relevant keywords: (‘acute myeloid leukemia’ OR ‘AML’) AND (‘homo sapiens’ OR ‘human’). The selection criteria were the following: (i) gene expression data derived from human AML patient samples and corresponding normal tissue samples, (ii) inclusion of more than 5000 genes in the dataset, and (iii) data from animal models or cell lines were excluded. In this way, two eligible datasets were selected.

The GEO series GSE9476 [95] (Table S1) contains genome-wide gene expression by microarray of blast cells from AML patients and (a) unselected bone marrow (BM), and (b) unselected peripheral blood (PB) samples from healthy donors. The Affymetrix Human Genome U133A Array [HG-U133A] platform was employed.

In GSE138702 [96] (Table S1), global gene expression analysis was performed using RNA-seq of total RNA isolated from the bone marrow of AML patients and matched healthy donors. The Illumina HiSeq 3000 (Homo sapiens) GPL21290 platform was used.

#### 4.6.1. Microarray-Based Transcriptomic Data Analysis

For microarray data analysis, the two-sample *t*-test was employed to identify genes differentially expressed in two groups: (i) leukemic blast cells from AML (*n* = 26) versus BM (*n* = 10), and (ii) AML (*n* = 26) versus PB (*n* = 10) (Table S1), by applying a methodology described in detail in previous studies [97,98]. The Benjamini–Hochberg method [85] for controlling FDR was applied; an FDR-adjusted *p* value less than or equal to 0.01 was considered as statistically significant in this study. For all statistical analyses, the Stata 13 statistical software package (https://www.stata.com/, accessed on 10 March 2021) [99] was used.

#### 4.6.2. RNA-seq-based Transcriptomic Data Analysis

The gene expression profiles of the bone marrow of 9 AML patients versus 10 healthy control donors were compared (Table S1). For the RNA sequencing analysis, the following pipeline was performed. FASTQ files that contained raw 2 × 50 bp paired-end RNA-seq reads were downloaded from the respective Sequence Read Archive (SRA) by using the SRA Tool Kit v.2.9.0 [100]. Raw RNA-seq reads were mapped to the human reference genome GRCh38 (Ensembl version 97) by employing the splice junction aligner HISAT2 v.2.1.0 [101]. Transcriptome normalization, reconstruction, and quantification were conducted by using StringTie v.1.3.5 [102]. Differentially expressed genes (DEGs) were detected using the edgeR package version 3.32.0 [81], by setting |log_2_FC ≥ 2| and the FDR-corrected *p* value less than 0.05.

### 4.7. Identification of DEGs Correlated with Long-Term Survival

Patient data from the TCGA LAML cohort were retrieved via the ‘TCGAbiolinks’ package (data.type: Gene Expression Quantification, workflow.type: HTSeq—Counts). Only the genes that were expressed in at least half of the patients were retained and the outliers that were >6 standard deviations away from the median were removed. We defined the groups of LTS patients (2 × SD = 1567 days, 3 × SD = 2168 days, and 5-year survival = 1825 days) and calculated the respective differentially expressed genes (DEGs) in the population that died within 365 days using edgeR [81], with log_2_FC cutoff > 1. Afterwards, we performed gene set enrichment analysis (GSEA) on the DEGs for each comparison, as well as for the common 131 genes that were deregulated in all three analyses. For GSEA, the GSEA-P 2.0 software (Broad Institute, Cambridge, MA, USA) [103] was used. Enriched Gene Ontology (GO) terms were plotted against the negative log10 of their individual FDR value (<0.05).

## 5. Conclusions

In conclusion, we applied in silico workflows on publicly available AML-relevant transcriptomic data and identified 24 genes mainly related to cell cycle and immune response, several of which were subsequently verified in two independent datasets. These genes could be extrapolated for the establishment of powerful AML diagnostic biomarkers and also be considered for defining patients that would most benefit from immunotherapeutics. Moreover, taking advantage of the fact that transcriptomic data of AML cases with unusually extended survival periods already exist in well-established databases, we tested a novel notion which proposes that rare patients at the extremes can be informative for prognosis and management of the general cancer population. Comparisons of the transcriptomes of these exceptional cases with those of more typical disease trajectories in our study cohort revealed an association of deregulations of nervous system-related genes with favorable AML prognosis. Future experimental and clinical studies can provide further insights on the underlying molecular mechanisms, inform clinical decisions, and catalyze the translation of these findings to diagnostic and therapeutic solutions.

## Data Availability

No new data were generated or analyzed in this study. Data sharing is not applicable to this article.

## Supplementary Materials

The following are available online at https://www.mdpi.com/article/10.3390/ijms22179601/s1.
