# Supplementary figures and images for: In Silico Methods for the Identification of Diagnostic and Favorable Prognostic Markers in Acute Myeloid Leukemia
Source: Int J Mol Sci. 2021 Sep 5;22(17):9601. doi: 10.3390/ijms22179601 (PMC8431757; doi:10.3390/ijms22179601)

## Slide 1
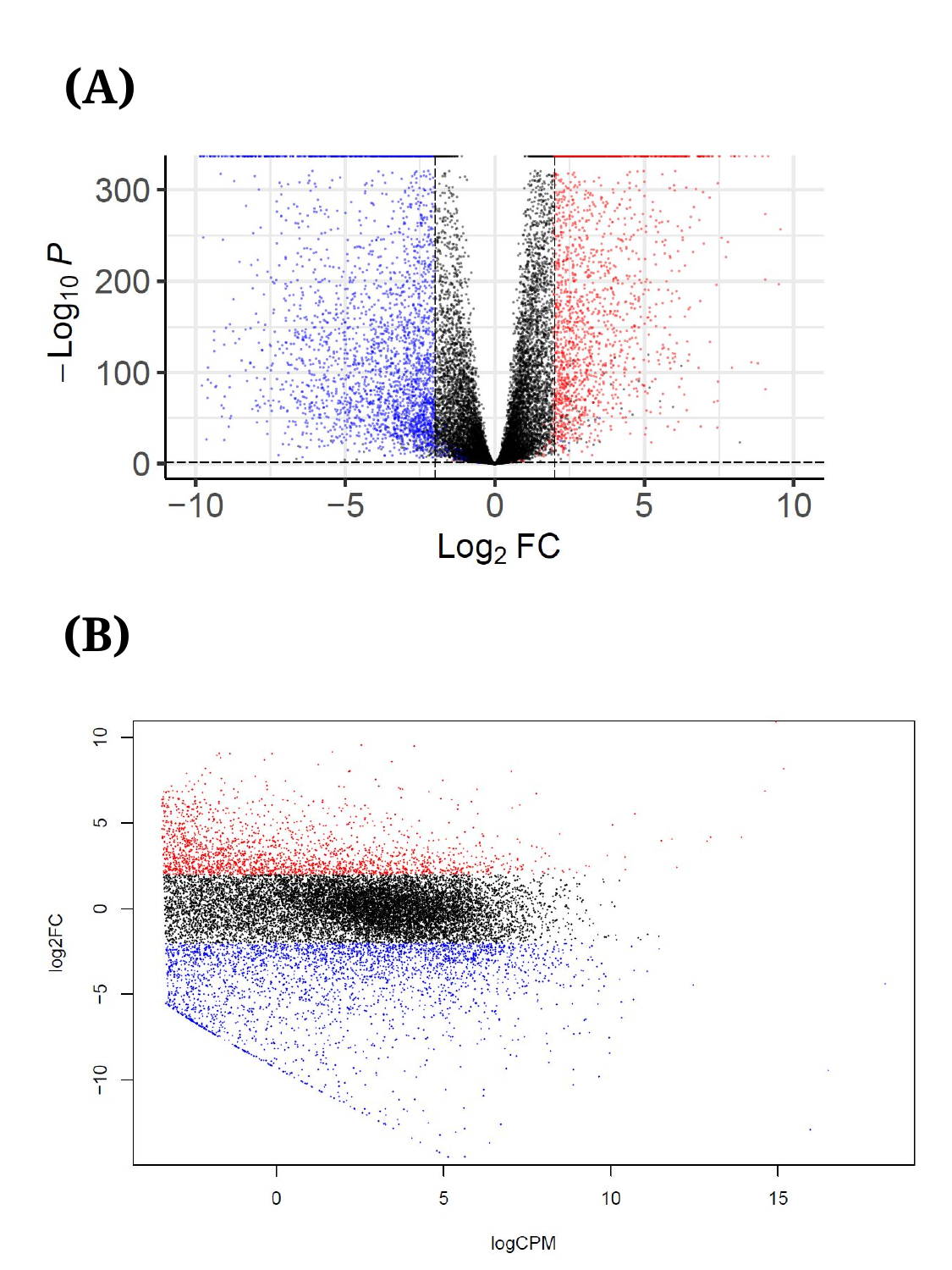

(A)
(B)

Supplement: Supplementary file 1 [file ijms-22-09601-s001.zip › Figure S1.pptx]
